# Rape on an Idiot

**Published:** 1847-01

**Authors:** 


*Rape on an Idiot.* Reg. v. Ryan.—The prisoner was indicted for rape. The prosecutrix was an idiot, and when asked questions in the witness-box was evidently unconscious of their purport, and not in a condition to understand right from wrong. Platt, B., interrogated her father as to her general habits, whether they were those of decency and propriety, and an answer in the affirmative was returned.

Platt, B. in summing up. The question is, did the connection take place with her consent? It seems that she was in a condition incapable of judging, and it is important to consider whether a young person, in such a state of incapacity, was likely to consent to the embraces of this man; because if her habits, however irresponsible she might be, were loose and indecent, there might be a probability of such consent being given, and a jury might not think it safe to conclude that she was not a willing party. But here the presumption is that the young woman would not have consented; and if she was in a state of unconsciousness at the time the connection took place, whether it was produced by any act of the prisoner, or by any act of her own, any one having connexion with her would be guilty of rape. If you believe that she was in a state of unconsciousness, the law assumes that the connection took place without her consent, and the prisoner is guilty of the crime charged. The prisoner was convicted. —*Ib. from Law Times.*

